# Development of GBTS and KASP Panels for Genetic Diversity, Population Structure, and Fingerprinting of a Large Collection of Broccoli (*Brassica oleracea* L. var. *italica*) in China

**DOI:** 10.3389/fpls.2021.655254

**Published:** 2021-06-04

**Authors:** Yusen Shen, Jiansheng Wang, Ranjan K. Shaw, Huifang Yu, Xiaoguang Sheng, Zhenqing Zhao, Sujuan Li, Honghui Gu

**Affiliations:** ^1^Institute of Vegetables, Zhejiang Academy of Agricultural Sciences, Hangzhou, China; ^2^Central Laboratory of Zhejiang Academy of Agricultural Sciences, Hangzhou, China

**Keywords:** broccoli, whole-genome sequencing, GBTS, KASP, genetic diversity, population structure, fingerprinting

## Abstract

Broccoli (*Brassica oleracea* var. *italica*) is one of the most important and nutritious vegetables widely cultivated in China. In the recent four decades, several improved varieties were bred and developed by Chinese breeders. However, the efforts for improvement of broccoli are hindered by limited information of genetic diversity and genetic relatedness contained within the available germplasms. This study evaluated the genetic diversity, genetic relationship, population structure, and fingerprinting of 372 accessions of broccoli representing most of the variability of broccoli in China. Millions of SNPs were identified by whole-genome sequencing of 23 representative broccoli genotypes. Through several stringent selection criteria, a total of 1,167 SNPs were selected to characterize genetic diversity and population structure. Of these markers, 1,067 SNPs were genotyped by target sequencing (GBTS), and 100 SNPs were genotyped by kompetitive allele specific PCR (KASP) assay. The average polymorphism information content (PIC) and expected heterozygosity (gene diversity) values were 0.33 and 0.42, respectively. Diversity analysis revealed the prevalence of low to moderate genetic diversity in the broccoli accessions indicating a narrow genetic base. Phylogenetic and principal component analyses revealed that the 372 accessions could be clustered into two main groups but with weak groupings. STRUCTURE analysis also suggested the presence of two subpopulations with weak genetic structure. Analysis of molecular variance (AMOVA) identified 13% variance among populations and 87% within populations revealing very low population differentiation, which could be attributed to massive gene flow and the reproductive biology of the crop. Based on high resolving power, a set of 28 KASP markers was chosen for DNA fingerprinting of the broccoli accessions for seed authentication and varietal identification. To the best of our knowledge, this is the first comprehensive study to measure diversity and population structure of a large collection of broccoli in China and also the first application of GBTS and KASP techniques in genetic characterization of broccoli. This work broadens the understanding of diversity, phylogeny, and population structure of a large collection of broccoli, which may enhance future breeding efforts to achieve higher productivity.

## Introduction

Broccoli (*Brassica oleracea* var. *italica*) is an economically important vegetable widely grown in many countries and is gaining popularity as a human diet due to its rich nutritional value as well as presence of anti-cancer glucosinolates in its florets (Wang et al., 2012). It was originated in the Mediterranean basin and introduced to China in the 20th century (Branca et al., 2005). In recent years, broccoli farming in China has made tremendous progress with about 80,000 ha of cultivated area in 2019. Additionally, many improved broccoli cultivars were produced and developed by Chinese breeders during the recent four decades, and China has become an important exporter of broccoli. However, most of these new cultivars are derived from a core collection of Japanese germplasm (Li et al., 2019) representing a narrow genetic background. For genetic improvement and conservation of crop species, it is vital to study the extent of genetic diversity and population structure prevailing in the species. Previously, only few studies were carried out to decipher the genetic diversity of broccoli using molecular markers (Tonguç and Griffiths, 2004; Lu et al., 2009; Huifang et al., 2011; Ciancaleoni et al., 2014; Stansell et al., 2018). However, a major concern is that, in most of the studies, there was poor representation of samples, or a limited number of samples was used raising the apprehension that the actual diversity present in broccoli has been underestimated. So, there is an urgent need to study the extent of molecular diversity present in broccoli to increase its productivity and quality. In the present investigation, a representative set of 372 broccoli accessions representing most of the variability were used for diversity analysis.

As the breeding of broccoli varieties are gaining momentum, many improved cultivars and modern varieties are being developed by both public and private organizations flooding the seed market. Consequently, it is difficult to distinguish the similar broccoli varieties in the seed trading market. The International Union for the Protection of New Varieties of Plants (UPOV) has established the “distinctness, uniformity, and stability” (DUS) testing for new varieties before registration (Jones et al., 2013). In this scenario, DNA fingerprinting can help in improving the knowledge of varieties, which are otherwise difficult to be distinguished phenotypically (Jones and Mackay, 2015). Additionally, DNA fingerprinting of crop species can also act as an insurance for the plant breeders to safeguard their important varieties and parental lines.

The molecular markers can assess the genetic diversity of crop species based on the variation of DNA that arises from substitution, insertion, and/or deletion in the chromosomes. Over the past three decades, several different DNA marker technologies, including amplified fragment length polymorphisms (AFLPs), simple sequence repeats (SSRs), single nucleotide polymorphisms (SNPs), and multiple nucleotide polymorphisms (MNPs) have been widely applied in DNA fingerprinting, genetic diversity, population structure analysis, and marker-assisted breeding (Tian et al., 2015). SSRs are routinely used for fingerprinting because of their high level of polymorphism (Rauscher and Simko, 2013; Pandino et al., 2015; Ouyang et al., 2018; Zheng et al., 2019). However, SSRs have some disadvantages; for instance, the throughput of detection is low, and the data integration or comparison between different detection platforms is difficult (Tian et al., 2015). On the contrary, SNPs are abundantly present in any given species (Deschamps and Campbell, 2010; Trebbi et al., 2011) and have been used in many genetic studies, including germplasm characterization (genetic diversity, genetic relationship, and population structure), quantitative trait loci (QTL) mapping, and marker-assisted selection (Ertiro et al., 2015). SNP array-based marker sets could also be used for fingerprinting (Tian et al., 2015; Thomson et al., 2017; Xu et al., 2017; Ellis et al., 2018), though this may not be their primary purpose. Recent emergence of next-generation sequencing technologies has made SNP discovery easy, rapid, cost effective, and with high throughput.

In recent times, several SNP genotyping platforms have been developed based on various technological principles, including TaqMan (Livak et al., 1995), genotyping by target sequencing (GBTS) (Guo et al., 2019), and kompetitive allele specific PCR (KASP) (KBiosciences^1^). GBTS is mainly divided into multiplex PCR-based target sequencing and probe-in-solution-based target sequencing. Nowadays, this platform contains several main technologies, such as AmpliSeq (Li et al., 2017), NimbleGen (Krasileva et al., 2017), SureSelect (Jiang et al., 2014), GenoBaits, and GenoPlexs (Guo et al., 2019). As a newly developed genotyping platform, GBTS combines the advantages of the fixed chips with the flexibility and low cost of GBS. As one of the targeted sequence-capture strategies, GBTS has been successfully utilized in maize (Guo et al., 2019), cucumber (Yang J. et al., 2019; Zhang et al., 2020), and pepper (Du et al., 2019). KASP assays developed by LGC Biosearch Technologies, United Kingdom, has emerged as a powerful tool and is based on allele-specific oligo extension and fluorescence resonance energy transfer (FRET) for signal generation and performs bi-allelic scoring of SNPs, insertions, and deletions (InDels) at specific loci (Hao et al., 2017). Compared with fixed chip array, KASP is a more flexible and cost-effective technology, especially for a small number of markers to genotype large number of samples. KASP assays have been used in various crops, such as rice (Steele et al., 2018; Yang G. et al., 2019), wheat (Rasheed et al., 2016), and maize (Ertiro et al., 2015). In some horticultural plants, KASP markers were used primarily for fine mapping of the candidate QTLs (Rett-Cadman et al., 2019; Zhu et al., 2019; Liao et al., 2020). To the best of our knowledge, the development of GBTS or KASP panels for diversity, relatedness, structure analysis, and fingerprinting studies has not been reported in broccoli.

In this backdrop, the present investigation was carried out with the following objectives: (i) to identify genome-wide SNPs through whole-genome sequencing (WGS) of a set of diverse genotypes of broccoli; (ii) to develop a GBTS platform suitable for genotyping broccoli accessions; (iii) to develop a KASP platform suitable for fingerprinting of broccoli accessions for varietal identification; (iv) to decipher the genetic diversity, genetic relationship, and population structure of 372 broccoli accessions.

## Materials and Methods

### Candidate Broccoli Genotypes for Sequencing

**FIGURE 1:**
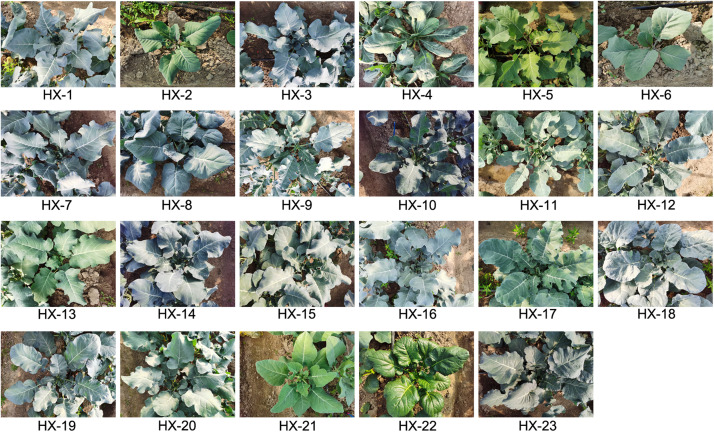
Twenty-three diverse broccoli lines selected for whole-genome sequencing.

Based on our previous study on phylogenetic relationship, a set of 23 diverse genotypes were selected for WGS because of their genetic distinctness. Among the 23 diverse broccoli genotypes, 10 were double haploid (DH) lines, and the others were advanced-generation inbred lines. These materials (Figure 1) are representative of many agronomical important traits of broccoli, such as head color, head shape, leaf angle, stem height, flowering time, etc. Detailed information of major morphological features (according to DUS) and source of these 23 diverse broccoli genotypes are given in Supplementary Table 1.

### Broccoli Accessions for Phylogenetic Study and Fingerprinting

To analyze the phylogenetic relationship, genetic variability, population structure, and also to carry out the fingerprinting for varietal identification, a set of 372 accessions were used. Most of these accessions (367) were provided by 11 broccoli breeding groups in China, and five hybrids (widely planted in China) were from the Sakata Seed Corporation of Japan. The 372 broccoli accessions comprised of 102 breeding lines (including DH lines, improved lines, and the parents of commercial hybrids), 248 landraces, and 22 commercial hybrids, could nearly cover all agro-ecological zones of China, where broccoli is widely cultivated.

### Whole-Genome Sequencing

Genomic DNA of 23 broccoli genotypes was extracted from young leaf tissues by a modified cetyl trimethyl ammonium bromide (CTAB) method (Hanania et al., 2004). The DNA quality and quantity were determined by a NanoDrop 2000 Spectrophotometer (Thermo Fisher Scientific, United States). The paired-end WGS libraries were constructed using Illumina’s sample preparation kit (Illumina Inc., United States) as per the manufacturer’s instructions. Genomic DNA (1 μg) was fragmented using Covaris S2 System (Covaris, Inc., United States) followed by adaptor ligation using the Kapa Hyper Prep kit (Kapa Biosystems). The ligated products were purified, and the enrichment of adaptor-modified fragments was performed by PCR. The profiles of the enriched libraries were assessed for size and quality using a high-sensitivity lab chip kit on an Agilent Bioanalyzer (Agilent Technologies, United States). A paired-end cluster generation kit (Illumina Inc., United States) was used for cluster generation on paired-end flow cells. Cluster generation was carried out by hybridization of WGS libraries with a concentration ranging from 10 to 40 nmol/μl, which again diluted to a stock solution of 4 nmol of molecules on the oligonucleotide-coated surface of the flow cell. Isothermal amplification of the libraries was carried out to generate clonal DNA clusters, and the libraries were sequenced on an Illumina HiSeq2500 instrument (Illumina, United States) with an average read length of 2 × 250 bp.

### Discovery of Genome-Wide Single Nucleotide Polymorphisms

The original image data generated by the Illumina sequencing machine were converted into sequence data via base calling (Illumina pipeline CASAVA v1.8.2), and the raw reads were then subjected to quality control (QC) procedure and adapter trimming to remove the unusable reads. The available draft genome sequence of HDEM broccoli with a genome size of 630 Mb (Belser et al., 2018) was downloaded, and the filtered sample reads were mapped to the reference genome using the BWA software (Li and Durbin, 2009) with default parameters. SNP discovery and filtration were carried out using SAMtools v0.1.19 (mpileup and varFilter) (Li et al., 2009) with default parameters. The raw SNP sets were called by SAMtools with the parameters as “-q 1 -C 50 -m 2 -F 0.002 -d 1000.” This set of SNPs was filtered by using the following criteria: (1) The mapping quality >20; (2) the depth of the variate position >4; (3) window size for occurrences of one SNP: 100 bp. The software ANNOVAR was used for functional annotation of variants (Wang et al., 2010), and the UCSC known genes were used for gene and region annotations (Hsu et al., 2006).

### Selection of Single-Nucleotide Polymorphisms for the Genotyping by Target Sequencing Platform and Genotyping

After SNPs were called for 23 broccoli genotypes, the quality of each SNP was assessed based on minor allele frequency (MAF) and polymorphic information content (PIC) using PowerMarker v3.25 software (Liu and Muse, 2005). Several filtering steps were performed to carefully select the SNPs for the GBTS platform. The filtration of SNPs was carried out by using the following criteria: (1) select the SNPs having missing data points of <5%; (2) SNPs with the MAF values of >0.2; (3) merge the adjacent SNPs to get MNPs, and the regions for each MNP should be less than 150 bp within more than one SNP located.

The GBTS library construction consisted of two rounds of PCR. In the first round, the target SNPs in plant DNA samples were amplified and captured using a multiplexed PCR panel. In the second round, a unique barcode was added to the captured product for each DNA sample. First, the multiplexed PCR was performed in 30-μl reactions including 20 ng of DNA template, 10 μl of GenoPlexs 3 × T Master Mix (Molbreeding Biotechnology Company, Shijiazhuang, China). The PCR conditions were as follows: 95°C for 5 min, then 16 cycles of 95°C for 30 s and 60°C for 4 min, and an extension at 72°C for 5 min. The PCR products were purified by magnetic bead suspension and 75% alcohol. Second, PCR was conducted in 30-μl reactions consisting of 11 μl of purified PCR product from the first round, 10 μl of GenoPlexs 3 × T Master Mix, and 1 μl of sequencing connector with barcode sequence. The PCR conditions were as follows: 95°C for 5 min, then nine cycles of 95°C for 30 s, 60°C for 20 s, and 72°C for 30 s, and an extension at 72°C for 5 min. The second round of PCR products was purified with 100 μl of 75% alcohol and 23 μl of Tris-HCl buffer (10 mM, pH 8.0–8.5). Thereafter, the constructed library was sequenced using Illumina HiSeq X Ten platform.

To better characterize the 372 broccoli accessions, the genotyping data were further filtrated as follows: (1) removing the SNPs having missing data points of >20%, (2) removing SNPs with the MAF values of <0.05, and (3) removing the SNPs with the PIC values of <0.2.

### Design of Kompetitive Allele Specific PCR Assay and Genotyping

The available SNPs were filtered based on several criteria to choose the best loci for KASP marker development. The filtration of SNPs was carried out by removing (1) SNPs having missing data points of >20%, (2) SNPs with the MAF values of <0.05, (3) other variations located within a distance of 50 bp upstream and downstream of the target SNPs, and (4) SNPs with the PIC values of <0.2. Among the SNPs amenable to KASP assay development, SNPs were further selected for designing of KASP primers by (1) removing the primer sequence with GC content lower than 0.3, (2) based on the loci present within the exonic, intergenic, and upstream/downstream of the functional genes, and (3) loci selected based on physical position, which are evenly distributed across the chromosomes.

For the selected KASP loci, primers were designed, and genotyping of the 372 broccoli accessions was carried out. Basically, the KASP assay contains three components: KASP assay mix, KASP master mix, and template DNA. The KASP assay mix (72X) contains two different, allele-specific, competing forward primers with unique tail sequences at the 5′ end (allele-1 tail has FAM-labeled oligo sequence and allele-2 tail has HEX-labeled oligo sequence) and one common reverse primer. KASP master mix (2X) contains FAM and HEX-specific FRET (fluorescence resonance energy transfer) cassette, ROX passive reference dye, KASP Taq DNA polymerase (specially modified for allele-specific PCR), dNTPs, and MgCl_2_ in an optimized buffer solution, which is universal to every KASP genotyping assay. The genomic DNA was extracted from 100 mg of young leaf tissue from each broccoli accession by a modified cetyl trimethyl ammonium bromide (CTAB) method (Hanania et al., 2004). The DNA quality and quantity were determined by a NanoDrop 2000 Spectrophotometer (Thermo Fisher Scientific, United States), and DNA concentration of 10–30 ng/ml was used for KASP genotyping. The PCR amplification was performed in 96-well microplates containing a final reaction volume of 10.14 μl. The PCR mixture in each well contained 5 μl of 10 ng/μl of genomic DNA, 5 μl of 2X KASP master mix, and 0.14 μl of KASP assay mixture. After dispensing the reaction mixture of 10.14 μl into a 96-well microplate, PCR was carried out using the KASP thermal cycling program of three steps [Initial activation: 94°C for 15 min; 10 touchdown cycles at 94°C for 20 s, and 61–55°C for 60 s (dropping 0.6°C per cycle); and finally, 26 cycles at 94°C for 20 s and 55°C at 60 s]. After the completion of PCR, the microplate was read with FRET-capable plate detector (Molecular Devices, Sunnyvale, CA, United States) for fluorescence detection of PCR product. Furthermore, the fluorescence readings were analyzed using KlusterCaller^TM^ Version 3.4.1.36 software (LGC Biosearch Technologies, United Kingdom) for visualization of allelic discrimination of each genotype.

### Genetic Diversity and Population Structure Analysis

The allelic data of the 372 broccoli accessions genotyped by GBTS and KASP panel were integrated. All the SNP markers were analyzed, and various genetic diversity summary statistics including observed heterozygosity (Ho), expected heterozygosity (He) also called gene diversity (GD), minor allele frequency (MAF), and polymorphic information content (PIC) were measured using the PowerMarker software v3.25 (Liu and Muse, 2005). The genotyping data were used to calculate the pairwise genetic distance matrix and to construct a phylogenetic tree by the neighbor-joining method using MEGA v10.0.4 software (Kumar et al., 2018), with 1,000 bootstrap replications to understand the relationship among the genotypes. Principal component analysis (PCA) was performed using the prcomp function in R language (v4.0.2) to visualize the overall representation of diversity in broccoli accessions.

The population structure in the broccoli accessions was assessed to identify the optimal number of subpopulations. The STRUCTURE v2.3.4 software package (Pritchard et al., 2000; Hubisz et al., 2009) was used for structure analysis based on Bayesian clustering approach. To determine the population structure, 10 runs for each *K* value from 1 to 10 was performed using 10 iterations for each *K*. For each run, a burn-in period of 50,000 was used to minimize the effect of the starting configuration, which was followed by an additional 100,000 iterations using a model with admixture (genotype might have mixed ancestry) and correlated allele frequencies. The likelihood of optimal *K* value was calculated, and the *K* value corresponding to the highest likelihood was interpreted as the number of subpopulations in the samples. The most probable *K* value was determined from the uppermost level of population structure, detected using an *ad hoc* statistic Δ*K* based on the rate of change in the log probability of data between successive *K* values (Evanno et al., 2005). Using the web-based STRUCTURE HARVESTER v0.6.94 (Earl and VonHoldt, 2012), the *ad hoc* statistic Δ*K* was calculated.

### Population Differentiation and Genetic Diversity Indices

Analysis of molecular variance (AMOVA) was computed using GenAlEx v6.502 (Peakall and Smouse, 2012) to estimate the variance components among and within the subpopulations generated by STRUCTURE HARVESTER. Population differentiation test such as the Wright’s fixation index (Fst) (Wright, 1965), which measures the amount of genetic variance of the populations was estimated using GenAlEx v6.502, and significance was tested based on 1,000 bootstraps. Gene flow (Nm) among the population was calculated using the formula, Nm = 0.25 (1 - Fst)/Fst (Slatkin and Barton, 1989). Nei’s genetic distance and several other genetic indices such as number of loci with private allele, number of different alleles (Na), number of effective alleles (Ne), Shannon’s information index (I), observed heterozygosity (Ho), and expected heterozygosity (He) were also calculated using GenAlEx v6.502.

## Results

### Whole-Genome Sequencing and Discovery of Single-Nucleotide Polymorphisms

Twenty-three diverse broccoli genotypes were used for WGS, and paired-end sequencing libraries were generated for each sample. The libraries were of good quality according to sizing profiles generated by high-sensitivity lab chip kit (Agilent Technologies, United States), and the fragment size of the libraries ranged from 450 to 750 bp. Sequencing of these libraries in the Illumina HiSeq2500 instrument yielded 13.1 to 18.7 Gb of raw data per sample (Table 1). A total of 346.18 Gb of raw data with an average of 15 Gb were generated for 23 broccoli genotypes. After quality filtering and adapter trimming, 345.57 Gb of high-quality data were available for further processing (Table 1). On average, 99.8% of raw data were able to pass the filtration process indicating the high quality of sequencing.

**TABLE 1:** Statistics on whole-genome sequencing of 23 genotypes.

Genotypes	Number of raw reads	Total data (Gb)	Mapped reads	Alignment (%)	Average depth (×)
HX-1	97,192,414	14.60	96,008,663	98.78	26.81
HX-2	96,297,844	14.46	94,695,611	98.34	27.55
HX-3	91,090,564	13.68	89,886,851	98.68	25.45
HX-4	88,358,380	13.27	86,911,004	98.36	25.99
HX-5	88,810,900	13.34	87,126,622	98.10	25.55
HX-6	91,782,854	13.79	90,186,403	98.26	26.09
HX-7	101,924,026	15.31	100,625,993	98.73	28.57
HX-8	100,947,244	15.16	99,489,024	98.56	27.92
HX-9	90,268,680	13.55	89,119,556	98.73	25.21
HX-10	95,782,732	14.38	94,745,032	98.92	26.33
HX-11	104,574,752	15.70	103,463,996	98.94	28.30
HX-12	107,531,638	16.15	106,399,003	98.95	29.43
HX-13	120,392,562	18.08	118,738,641	98.63	32.79
HX-14	100,768,972	15.13	99,663,458	98.90	28.48
HX-15	98,685,420	14.82	97,357,254	98.65	27.40
HX-16	104,594,330	15.71	102,981,890	98.46	29.10
HX-17	113,117,330	16.99	111,587,466	98.65	31.07
HX-18	124,943,550	18.77	123,081,201	98.51	34.59
HX-19	94,680,260	14.22	93,710,734	98.98	25.77
HX-20	96,140,460	14.45	94,838,250	98.65	27.65
HX-21	100,730,344	15.14	99,606,256	98.88	28.38
HX-22	87,695,482	13.19	86,434,061	98.56	25.07
HX-23	107,536,214	16.18	106,246,250	98.80	30.15
Average	100,167,259	15.05	98,821,879	98.65	27.98

The number of reads per sample ranged from 87.7 to 124.9 million (Table 1). The Hiseq sequencing platform yielded a total of 2,303.9 million paired reads with an average of 100 million reads. The high-quality filtered reads were mapped to the broccoli reference genome. Out of a total of 2,303.9 million reads, 2,272.9 million reads were successfully aligned to the broccoli reference genome. The mapping percentage of each sample ranged from 98.1% to 98.98%. On average, 98.65% of the reads could be successfully mapped to the reference genome. The sequence depth of mapping reads ranged from 25 to 34.59× with an average of 28× (Table 1). A million numbers of SNPs were detected between the sample and reference genome ranging from 899,926 to 1,908,908 (Table 2). Among the identified SNPs, the number of transitions (Ti) was more than transversions (Ts) across the samples, and the average Ti/Ts was found to be 1.43. The intergenic SNPs were almost more than double the number of genic SNPs (Table 2).

**TABLE 2:** Statistics on single nucleotide polymorphisms (SNPs) identified between the samples and the reference.

Genotypes	Total SNPs identified	Transition (Ti)	Trans version (Ts)	Ti/Ts	Genic SNPs	Intergenic SNPs
HX-1	1,158,081	681,828	476,253	1.43	265,391	669,826
HX-2	1,738,501	1,022,217	716,284	1.43	403,819	990,748
HX-3	1,201,122	705,991	495,131	1.43	265,879	708,587
HX-4	1,712,276	1,006,504	705,772	1.43	396,290	980,145
HX-5	1,908,908	1,121,167	787,741	1.42	418,966	1,125,604
HX-6	1,707,755	1,002,927	704,828	1.42	398,259	972,794
HX-7	1,192,357	701,175	491,182	1.43	260,217	708,394
HX-8	1,448,231	851,295	596,936	1.43	318,736	856,671
HX-9	1,201,811	707,932	493,879	1.43	270,738	702,699
HX-10	946,171	557,839	388,332	1.44	211,313	555,371
HX-11	991,691	585,706	405,985	1.44	227,813	573,127
HX-12	970,147	571,855	398,292	1.44	215,391	570,691
HX-13	1,321,936	778,187	543,749	1.43	283,100	790,635
HX-14	1,066,173	626,568	439,605	1.43	244,623	618,534
HX-15	1,205,647	709,834	495,813	1.43	268,203	711,509
HX-16	1,415,974	832,819	583,155	1.43	310,570	838,402
HX-17	1,291,865	759,962	531,903	1.43	288,518	759,049
HX-18	1,422,938	838,731	584,207	1.44	313,859	836,882
HX-19	899,926	529,813	370,113	1.43	218,272	504,722
HX-20	1,421,281	835,484	585,797	1.43	302,010	857,048
HX-21	1,163,649	684,612	479,037	1.43	259,640	683,677
HX-22	1,289,277	757,110	532,167	1.42	289,306	760,051
HX-23	1,206,976	711,004	495,972	1.43	280,159	693,236
Average	1,299,248	764,372	534,875	1.43	291,786	759,496

### Genotyping by Target Sequencing and Kompetitive Allele Specific Panel Development

The putative SNPs identified through the re-sequencing of 23 broccoli genotypes were further filtered for the GBTS and KASP panel developments, respectively.

**FIGURE 2:**
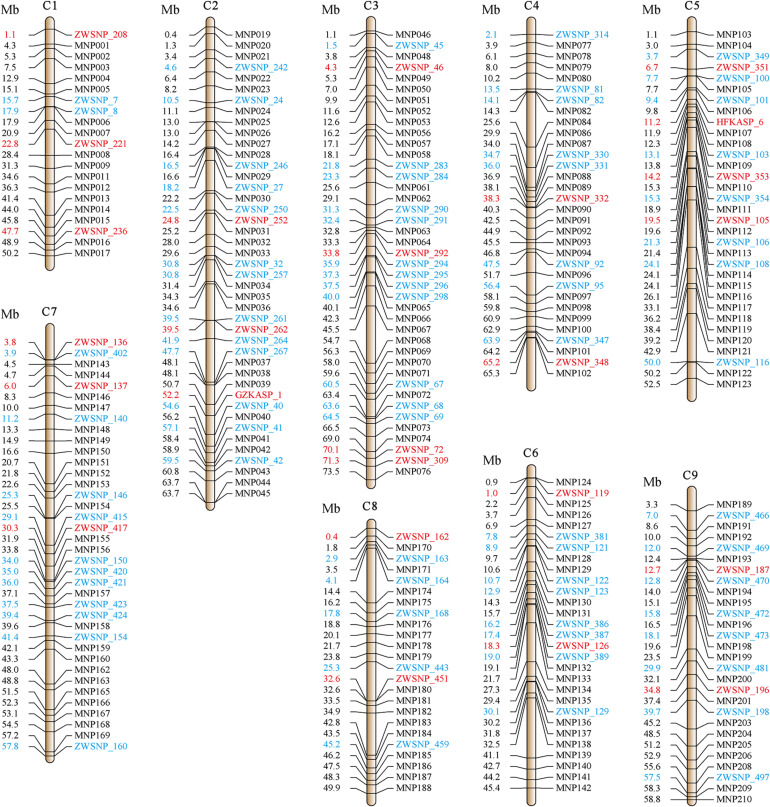
Distribution of all markers among broccoli chromosomes. The horizontal lines perpendicular to a chromosome represent the markers developed in this study, among which the black ones were developed by the GBTS panel, the blue and red ones were developed by the KASP panel, and the red ones were core KASP markers selected for fingerprinting of broccoli accessions.

For the GBTS panel, 210 MNPs consisting of 1,332 SNPs were selected, with an average of 6.3 SNPs within each MNP. The utility of these SNPs in deciphering the genetic relationship and diversity of 372 broccoli accessions was assessed by calculating the missing data, MAF, and PIC values. As a result, 1,067 (80.1%) SNPs were retained for further analysis (Supplementary Table 2). These SNPs were located in 189 MNP regions and evenly distributed across the nine chromosomes (Figure 2).

**FIGURE 3:**
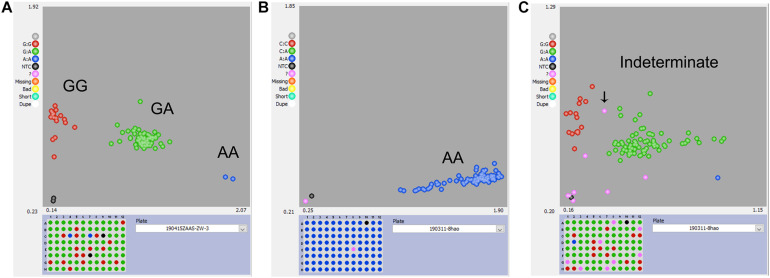
Representative results of KASP genotyping assay. **(A)** KASP marker with good polymorphism that was successfully developed. **(B)** KASP marker with no polymorphism that should be discarded. **(C)** KASP marker with more than 10% missing values or had ambiguous SNP-calling that should be discarded.

For the KASP panel, a total of 13,621 SNPs with the PIC values between 0.2 and 0.5 were retained after filtration. Of these, only 8,768 (64.4%) SNPs could be successfully designed as KASP markers. From this set, 2,515 SNPs were from exonic (including non-synonymous, stop-gain and stop-loss SNPs), intronic, intergenic regions, and from the upstream or downstream of the functional genes. Based on their functional role in regulating important agronomic traits and also based on the physical position and uniform genomic distribution, finally, 500 SNPs were selected for KASP marker development. Of the targeted 500 KASP markers, only 347 (69.4%) could be genotyped successfully with high-quality genotype clusters (Figure 3A), while clear clustering was not obtained for the remaining KASP markers. Out of 347 scorable KASP markers, 54 KASPs were monomorphic (Figure 3B), so the remaining 293 KASP markers were used for genotyping of the 372 broccoli accessions. Based on the genotyping data, KASPs having missing data points of more than 10% or showing ambiguous SNP call (Figure 3C) were removed. Finally, 100 KASPs with clear genotype cluster and even representation of the chromosomes (Figure 2, Supplementary Table 2, and Supplementary Table 3) were called successfully with high confidence and selected for further analysis.

### Genotyping of 372 Broccoli Accessions for Assessment of Genetic Diversity

**FIGURE 4:**
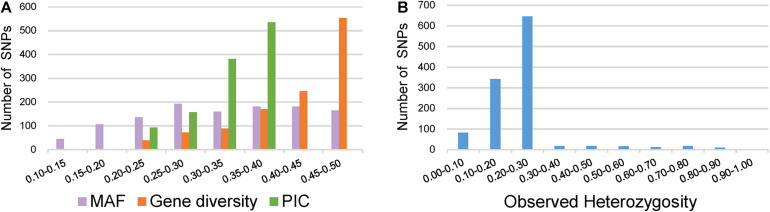
Distribution of genetic diversity for 1,167 markers in the 372 broccoli accessions. **(A)** Minor allele frequency (MAF), gene diversity (GD), and polymorphic information content (PIC). **(B)** Observed heterozygosity.

Key descriptive statistics for measuring the utility and informativeness of SNP markers were assessed by analyzing the genetic diversity of the 372 broccoli accessions (Supplementary Table 4). Of the 1,167 SNPs, 1,067 SNPs were from the GBTS panel, and 100 SNPs were from the KASP panel. The MAF values of all SNPs were 0.11–0.50, with a mean of 0.33 (Figure 4A). Most of the SNPs (87%) have MAF values more than 0.2, which could be considered as markers with normal allele frequency. Interestingly, not a single SNP had MAF of <0.05. The PIC values of the individual SNPs varied and ranged from 0.18 to 0.37 with a mean value of 0.33 (Figure 4A). The maximum percentage of SNPs (46%) was having PIC values ranging from 0.35 to 0.37. The observed heterozygosity for the KASP marker ranged from 0.00 to 0.93 with an average of 0.23 (Figure 4B). The expected heterozygosity (gene diversity) ranged from 0.20 to 0.50 with an average of 0.42 (Figure 4A).

### Genetic Relationship and Population Structure

The pairwise genetic distance matrix ranged from 0.0004 to 0.6418 with a mean of 0.3894 (Supplementary Table 5), indicating the presence of considerable diversity in the broccoli accessions, though few were closely related.

**FIGURE 5:**
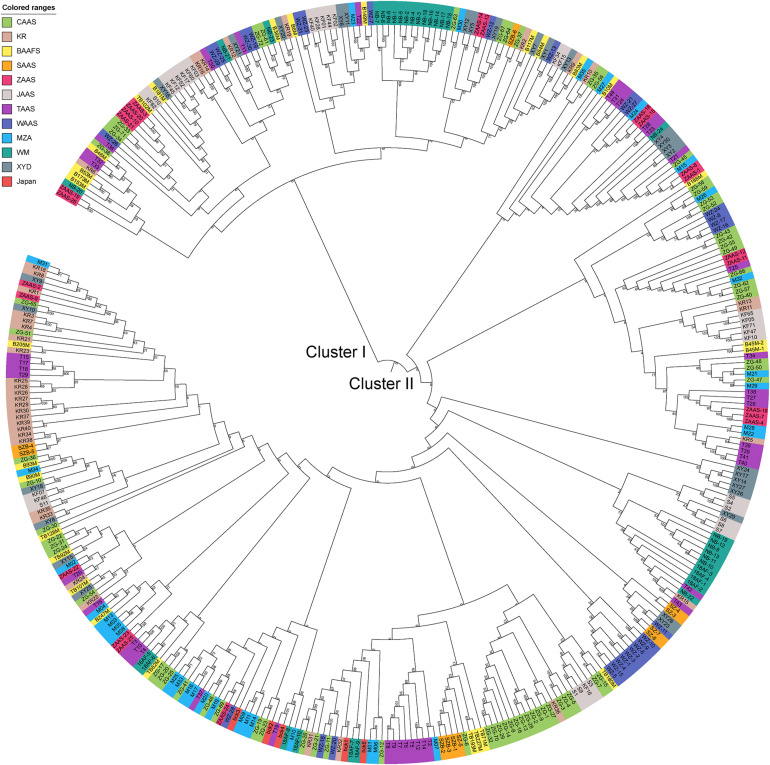
Phylogenetic tree constructed using the neighbor-joining method based on 372 broccoli accessions. The colors in outer circle represent different sources of the accessions. CAAS, Chinese Academy of Agricultural Sciences; KR, Tianjin Kerun Vegetable Research Institute; BAAFS, Beijing Academy of Agriculture and Forestry Sciences; SAAS, Shanghai Academy of Agricultural Sciences; ZAAS, Zhejiang Academy of Agricultural Sciences; JAAS, Jiangsu Academy of Agricultural Sciences; TAAS, Taizhou Academy of Agricultural Sciences; WAAS, Wenzhou Academy of Agricultural Sciences; MZA, Zhejiang Mitsuo Seed Co., Ltd.; WM, Ningbo Weimeng Seed Co., Ltd.; XYD, Zhenjiang Xinyuanda Horticulture Technology Co., Ltd.; Japan, Sakata Seed Corporation, Japan.

**FIGURE 6:**
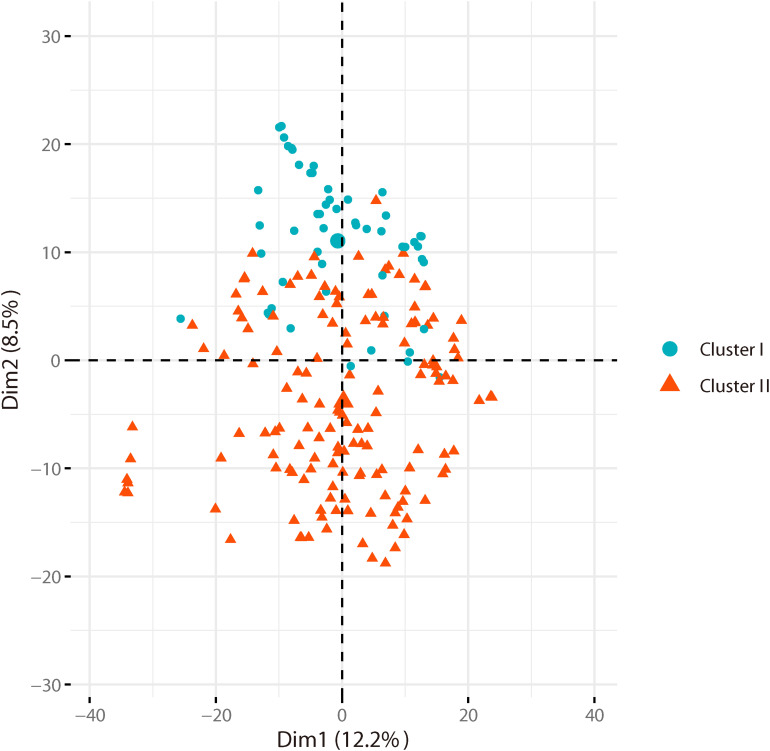
Principal component analysis (PCA) of broccoli accessions. Axes-1 (12.2%) and Axes-2 (8.5%) separate the genotypes into two groups.

Based on the genotyping data of 1,167 SNP markers in 372 broccoli accessions (Supplementary Table 3), a phylogenetic tree was constructed by the neighbor-joining method using the MEGA software. Cluster analysis revealed that all the 372 broccoli accessions were clustered into two major groups (Figure 5) with many sub-groups within the major groups, but importantly, the grouping was not supported by high bootstrap values, indicating weak grouping of broccoli accessions. The phylogenetic tree depicting the genetic relationship of 372 broccoli accessions were as expected based on the breeding history, and accessions sharing the pedigree were placed in close proximity. The cluster I containing 98 accessions were mostly improved varieties, and the cluster II contained 274 accessions, which were mostly landraces. PCA based on the pairwise genetic distance matrix complemented the cluster analysis results, and the samples were grouped into two clusters (Figure 6). The first and second axes of PCA captured only 8.5% and 12.2% of the overall variance, respectively. This again explained the weak grouping of the broccoli accessions in the present study.

**FIGURE 7:**
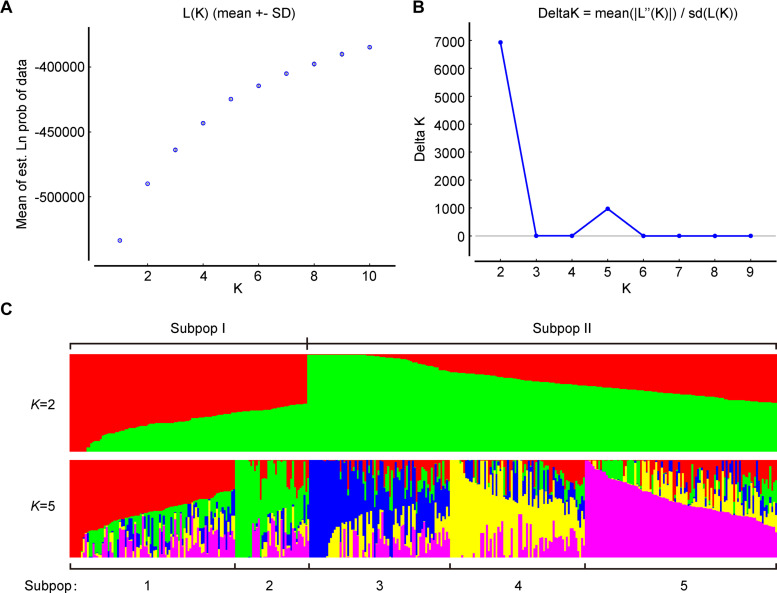
Population structure of 372 broccoli accessions based on 1,167 markers. **(A)** Log probability of data, L(*K*) averaged over the replicates. **(B)**Δ*K* values plotted as the number of subpopulations. **(C)** Subpopulations (*K* = 2 and *K* = 5) inferred using structure analysis.

To further verify the results of phylogenetic and PCA analyses, the population structure of 372 broccoli accessions were studied by STRUCTURE v2.3.4 (Pritchard et al., 2000; Hubisz et al., 2009). The number of clusters (*K*) of the accessions was estimated by setting the number of clusters (*K*) from 1 to 10 with 10 replications for each *K*. The average logarithm of the probability of likelihood [LnP(D)] and standard deviations for different number of sub-populations (*K* = 1 to 10) are presented in Supplementary Table 6. The most likely number of clusters (*K*) was selected by comparing the logarithmized probabilities of the LnP(D) and Δ*K* data (Yang G. et al., 2019). In this study, the LnP(D) showed continuous gradual increase with the increase in *K* (Figure 7A), making it difficult to assume the best value of *K*. Remarkably, the number of clusters (*K*) was plotted against Δ*K*, which showed a sharp peak at *K* = 2 (Figure 7B). Hence, based on the *ad hoc* statistic Δ*K* method, we decided to choose the value of *K* = 2 for our analysis, which clearly indicated the presence of two subpopulations within the broccoli accessions. A total of 125 accessions were assigned to subpopulation-I and 247 accessions to subpopulation-II (Figure 7C and Supplementary Figure 1). The bar plot also exhibited wide admixture in the broccoli accessions (Supplementary Figure 1), and the two subpopulations did not show any association with the geographic origin of the materials. The STRUCTURE result was in accordance with the results of the phylogenetic and the principal component analyses, which also showed the presence of weak grouping, indicating extensive exchange of the broccoli accessions by breeders. In addition, there was a small peak observed at *K* = 5 (Figure 7C), which might indicate another informative population structure. Therefore, the STRUCTURE results at both *K* = 2 and *K* = 5 were subject to the following population genetic analyses.

### Genetic Differentiation of Population and Gene Flow

To estimate the genetic variation among and within the two subpopulations identified in STRUCTURE, analysis of molecular variance (AMOVA) was carried out using GenAlEx v6.502. The *F*_*ST*_ value among the two populations (POP1 and POP2) was 0.126, supporting low population differentiations. The population differentiation based on AMOVA revealed that only 13% (*p* < 0.001) of the total variation was found among the subpopulations, while the rest (87%) was within the populations (Supplementary Figure 2). In addition, the overall gene flow among the population was estimated to be high (Nm = 1.741) (Table 3). Nei’s standard genetic distance between the two subpopulations was 0.103. The AMOVA and pairwise population *F*_*ST*_ analyses were done based on the population structure at *K* = 5, and the results are shown in Supplementary Tables 7, 8.

**TABLE 3:** Analysis of molecular variance (AMOVA) depicting the genetic variation among and within two subpopulations of broccoli.

		df	SS	MS	Est. Var.	%
**Source**	**Among populations**	1	11,370.844	11,370.844	33.547	13%
	**Within populations**	742	173,385.349	233.673	233.673	87%
	**Total**	743	184,756.194		267.220	100%
**F-statistics**	**Fst**	0.126				
	**Nm**	1.741				

### Allelic Pattern in the Subpopulations

The subpopulation I (I = 0.596, He = 0.411 and uHe = 0.413) shows higher diversity than the subpopulation II (I = 0.570, He = 0.388 and uHe = 0.388) (Table 4). The percentage of polymorphic loci per population (PPL) for the two populations was 100%. The grand mean value of different alleles (Na) and number of effective alleles (Ne) of the two subpopulations were 1.740 and 1.676, respectively. The grand mean value of I, He, and uHe for the two populations were 0.583, 0.399, and 0.401, respectively (Table 4).

**TABLE 4:** Mean of different genetic parameters in each of the two subpopulations.

Population	N	Na	Ne	I	Ho	He	uHe	F	PPL
Pop1	124.675	2.000	1.740	0.596	0.247	0.411	0.413	0.394	100.00%
Pop2	246.045	2.004	1.676	0.570	0.223	0.388	0.388	0.427	100.00%
Mean	185.360	2.002	1.708	0.583	0.235	0.399	0.401	0.411	100.00%

*N, the number of samples; Na, number of different alleles; Ne, number of effective alleles = 1/(Sum p_*i*_^∧^2); I, Shannon’s Information Index = -1 × Sum[p_*i*_ × Ln (p_*i*_)]; Ho, observed heterozygosity = number of Hets/N; He, expected heterozygosity = 1 - Sum p_*i*_^∧^2; uHe, unbiased expected heterozygosity = [2N/(2N - 1)] × He; F, Fixation Index = (He - Ho)/He = 1 - (Ho/He); PPL, percentage of polymorphic loci, where p_*i*_ is the frequency of the ith allele for the population and Sum p_*i*_^∧^2 is the sum of the squared population allele frequencies.*

### Selection of Core Kompetitive Allele Specific PCR Markers for Fingerprinting of Broccoli Accessions

To build a rapid and cost-effective way of varietal identification, we selected 28 KASP markers from the genotyping database for the fingerprinting of every accession (Supplementary Tables 2, 3). These KASP markers were highly effective in distinguishing the 372 examined accessions, as the markers are evenly distributed across the nine chromosomes (Figure 2). For each accession, the genotype-based KASP barcode was used to generate a corresponding 2D barcode using an online tool^2^. This barcode can be scanned to obtain the information used for creating the 2D barcode. Supplementary Figure 3 depicts a barcode of a representative variety of broccoli used in the present study.

## Discussion

### Whole-Genome Sequencing and Single-Nucleotide Polymorphism Discovery

In the current study, Illumina platform was chosen to undertake the whole-genome sequencing of 23 diverse broccoli genotypes and discover genome-wide SNPs. The Illumina platform was preferred for WGS due to its throughput, cost effectiveness, and indexing capabilities. The WGS data were generated with a high coverage (approximately 28×), and a total of 346.18 Gb of raw data for 23 broccoli genotypes were generated. Sequencing with high coverage gives confidence for SNP calling as low coverage sequencing will make SNP calling difficult with high genotyping errors (Heffelfinger et al., 2014). The raw data were of high quality as, on average, more than 99% were able to pass the filtration process with an error rate of as low as 0.03.

The samples reads were aligned to the reference genome of broccoli (HDEM), and the alignment percentage ranged from 98.1 to 98.98 with an average of 98.65%. The high mapping percentage signifies high quality of the sample reads. Among the SNPs, transition SNPs were more frequent than the transversions, which is a common phenomenon in *Brassica* species (Park et al., 2010; Huang et al., 2013). This happens due to synonymous mutations in protein-coding sequences (Guo et al., 2017) and suggested that transition mutations are better tolerated than transversion mutations during natural selection (Luo et al., 2017). The average transition/transversion ratio was observed to be slightly lower (1.43) indicating a high level of sequence divergence (Yang and Yoder, 1999). The genic SNPs identified were twofold lesser than the intergenic SNPs, and this could be possible as intergenic regions evolve faster and accumulate higher polymorphism than the genic regions, which are mostly conserved (Guo et al., 2007). The intergenic region could be a great source of SNPs for genetic studies, which has been poorly exploited in several crops.

### Genotyping by Target Sequencing and Kompetitive Allele Specific PCR Panel Development

In this study, a total of 1,067 markers were selected for the GBTS panel development. This GBTS panel with larger numbers of markers can effectively be used for genetic diversity and population structure analysis. In maize, a series of high-quality GBTS panels, including 20 K, 10 K, 5 K, and 1 K SNP loci were developed, making it an affordable genotyping platform for maize marker-assisted breeding (Guo et al., 2019). Based on the targeted sequence-capture strategy, 382 key cucumber varieties were genotyped by 122 SSRs using target SSR-seq method (Yang J. et al., 2019), and 261 cucumber varieties were genotyped by 163 SNPs using target SNP-seq method (Zhang et al., 2020). In pepper, 92 SNPs were used to detect polymorphisms across 271 commercial pepper varieties (Du et al., 2019). In the present study, 210 multiple-SNP (MNP) regions consisting of 1,332 SNPs were selected from the whole-genome sequencing of the 23 broccoli genotypes. A total of 372 broccoli accessions were genotyped by these SNPs, and 1,067 (80.1%) SNPs were selected for genetic diversity and population structure analysis of the broccoli accessions.

For the fingerprinting of the broccoli lines, few KASP markers will be more cost effective and flexible. KASP assay has been widely used by plant breeders for genetic diversity study, genetic mapping, and genetic purity test in several major crops including pigeon pea (Saxena et al., 2012), chickpea (Hiremath et al., 2012), maize (Jagtap et al., 2020), cotton (Islam et al., 2015), tomato (Devran et al., 2016), peanut (Zhao et al., 2017), wheat (Rasheed et al., 2016), and rice (Pariasca-Tanaka et al., 2015; Cheon et al., 2018; Steele et al., 2018; Yang G. et al., 2019). In the present study, out of 28,220 filtered SNPs, 13,621 SNPs with PIC values between 0.2 and 0.5 were retained for KASP assay design. However, only 8,768 (64.4%) SNPs could be designed as KASP markers successfully. This low conversion rate may have occurred probably due to the presence of duplicate loci, paralogous sequence, or incorrect primer design (Semagn et al., 2014; Steele et al., 2018; Jagtap et al., 2020). Optimizing the PCR conditions may improve the rate of successful KASP assay design.

### Assessment of the Genetic Diversity

A total of 372 broccoli accessions represents a wide variability and comprised of several improved lines from China, elite breeding lines, parents of commercial hybrids, landraces, and a few commercial hybrids. All these accessions were genotyped by 1,167 SNPs developed from the GBTS and KASP panels. The PIC values and expected heterozygosity (also called gene diversity) are two parameters in measuring genetic diversity in any population. In the present study, the PIC values of the SNPs ranged from 0.18 to 0.37 with a mean value of 0.33. In a previous study by Li et al. (2019) who evaluated the genetic diversity of 95 broccoli genotypes of China with SSR markers, the PIC values of the markers ranging from 0.48 to 0.99, with an average of 0.79, which was considerably high were found. The average PIC value of 0.33 in the present study could be considered high due to the bi-allelic nature of the SNPs, which restrict the range of PIC values from 0 to 0.5 (Eltaher et al., 2018). The expected heterozygosity (gene diversity) ranged from 0.20 to 0.50 with a mean value of 0.42. The mean value of expected heterozygosity revealed the prevalence of low to moderate level of diversity in broccoli. This study is comprised of broccoli accessions provided by 11 breeding groups in China, so good representation of diversity of broccoli was expected. The use of a representative set of germplasm is necessary to get the proper estimate of gene diversity in a species (Senthilvel et al., 2017). Though for the first time, a large collection of broccoli accessions with a representative set of accessions was used to estimate genetic diversity of broccoli in China, high level of diversity was not observed.

The MAF value of the SNPs ranged from 0.11 to 0.50, with a mean of 0.33. MAF threshold dramatically affects population structure inference, and inference of population structure is sensitive to MAF (Linck and Battey, 2019). SNPs with low MAF detected through NGS analysis tend to be less polymorphic than SNPs with higher MAF. In genetic diversity studies, it is desirable to maximize the number of polymorphic markers by selecting SNPs with moderate to high MAF. In this context, 87% SNPs have the MAF values more than 0.2, and importantly, not a single marker has MAF < 0.05. So, the set of SNP markers used in the present study is ideal for structure analysis and association studies.

### Genetic Relatedness and Structure

The pairwise genetic distance matrix ranged from 0.0004 to 0.6418 with a mean of 0.3894. Most of the accessions were found considerably distant, though a few accessions, mostly improved cultivars, were found genetically similar indicating the accessions having a common breeding history. Maximum genetic distance of 0.6418 was found between the broccoli accession ZAAS-8 and KR8. Interestingly, the genetic distance between several broccoli accessions belonging to different breeding groups was reported to be as low as 0.0004 (M31 and KR18). This could be due to the massive exchange of materials between different broccoli breeding groups in China resulting in the development of genetically similar broccoli cultivars. Recently, Li et al. (2019) reported high similarity coefficients ranging from 0.6909 to 0.8969 with an average of 0.7809 among the 95 broccoli genotypes in China collected from around the world. Lu et al. (2009) also reported a high similarity ranging from 0.8421 to 0.9330 between several broccoli genotypes. Several authors (Tonguç and Griffiths, 2004; Louarn et al., 2007) also reported similar close relationship of broccoli genotypes and suggesting the prevalence of narrow genetic base in broccoli (Hu and Quiros, 1991).

For phylogenetic analysis, all 372 broccoli accessions were clustered into two major groups with many sub-groups within the major groups, but the grouping was not supported by high bootstrap values indicating the prevalence of weak grouping patterns. Low bootstrap values suggest recombination or gene flow between different “branches” of the phylogenetic tree. Again, low bootstrap values explain that the members on the branch should not be divided into separate groups as it seems. The accessions that share the pedigree and were having similar breeding history were placed in close proximity as expected. Group I contained 98 accessions and comprised of improved varieties. Group II contained 274 accessions and represents mostly landraces and few improved accessions. Five Japanese F_1_ hybrids were grouped in cluster II and were placed closely with several accessions such as M14, T19, and KR 32. As mentioned earlier, broccoli was introduced into China from Japan four decades back (Li et al., 2019). Most of the modern broccoli cultivars of China might have been derived from the Japanese lines. So not surprisingly, the genetic distance between the Japanese F_1_ hybrids used in the present study and several improved varieties were found to be very low (Supplementary Table 5). The phylogenetic analysis again confirms the lineage of the Chinese broccoli varieties to the Japanese broccoli. Though from 1990 to the early 21st century, many broccoli cultivars were bred and developed by Chinese institutes (Li et al., 2019), still most of the broccoli cultivars widely planted in China are closely related and have a narrow genetic background. So, there is a necessity to broaden the genetic base of Chinese broccoli by incorporating diverse germplasm into the breeding program. However, the landraces used in the present study were genetically more diverse and could be used in the breeding program. PCA results corroborated with the findings of phylogenetic tree indicated that the samples were grouped into two clusters, but the variance captured by the first and second axes of PCA was only 8.5% and 12.2%, respectively, again explaining weak grouping.

All 372 broccoli accessions were divided into two sub-populations by the STRUCTURE analysis, which was in agreement with the phylogenetic tree and PCA results. Subpopulation-I contained 125 accessions, and subpopulation-II contained 247 accessions. Most of the broccoli accessions in cluster I of the phylogenetic tree were part of sub-population I. Similarly, most of the broccoli accessions in cluster II of the phylogenetic tree were placed in sub-population II (Supplementary Figure 1). On the whole, STRUCTURE and phylogenetic analyses agreed with each other with minor exceptions. This small discrepancy between the two methods of grouping is expected as the cluster analysis in neighbor joining tree assigned a fixed branch position to each accession, while STRUCTURE analysis resulted in a sub-population membership percentage, and the highest percentage was used to assign individuals to groups for easy interpretation (Wang et al., 2009), but importantly, the Fst value between the two populations was found to be low (0.126), suggesting the existence of weak genetic structure in the panel of 372 broccoli accessions. The two subpopulations also did not show any association with the geographic origin of the materials, indicating continuous exchange of broccoli parental lines among the breeders in China, and a close relationship exist among the broccoli accessions. Similar results of weak grouping coincided with the PCA and phylogenetic tree analyses. In the two inferred groups (Supplementary Figure 1), the samples were observed with potential admixture.

Population structure study is important for association mapping studies, and testing of population structure is conducted first to identify true marker–trait association. Absence of strong structure is a desirable feature for association analysis to avoid spurious marker–trait associations (Flint-Garcia et al., 2003). In this context, the panel of 372 broccoli accessions used in the present study is an ideal population for association study to identify associations between several agronomically important traits and marker alleles in broccoli. Furthermore, genetic diversity and population structure assessment study will assist the breeders in developing a core collection of broccoli for breeding and molecular studies.

### Genetic Differentiation of Populations

Fst value is a measure of population differentiation due to genetic structure. According to Wright (1943), the population differentiation could be considered high if the Fst value is greater than 0.25. The genetic differentiation between the two subpopulations was measured, and the overall Fst value was found to be low (0.126). Nei’s pairwise genetic distance among the two subpopulations was also found to be low (0.103). A low level of differentiation among the two subpopulations could have happened due to extensive gene flow, which acts as a powerful force to decrease population differentiation. Basically, gene flow value (Nm) greater than 1 is “strong enough” to prevent population differentiation (Slatkin and Barton, 1989). The low population differentiation of the present study was supported by sufficient gene flow (Nm = 1.741), suggesting a high genetic exchange between the populations. Low Fst value also coincided with AMOVA results where majority of the total variation (87%) was due to the “within the populations” variations, while only 13% of the total variation was due to “among-subpopulation” variations (Supplementary Figure 2). Majority of the genetic variations are harbored by individuals of the present study. These results again confirmed the finding of phylogenetic and STRUCTURE analysis, which showed the presence of weak genetic structure in the broccoli accessions.

### Fingerprinting of Broccoli Accessions

Due to a short breeding history of broccoli in China and also due to close relationship between certain varieties, it becomes difficult to distinguish between different cultivars using only morphological characters. Often, spurious seeds are easily mixed with the genuine ones in the broccoli seed market, which eventually harm the interest of consumers and breeders. Additionally, cytoplasmic male sterility (CMS) lines are widely used for the development of commercial F_1_ hybrids of broccoli. There is every chance that the CMS lines may get contaminated by foreign pollen, mechanical admixture, artificial mislabeling, or by mixing with other heterogeneous seeds during seed production. Loss of parental line purity can cause irreparable damage to the breeders. To solve these problems, it is essential to confirm the seed authenticity and purity of the parental lines before hybrid development and distribution.

In this context, DNA fingerprinting could act as an insurance for the breeders in safeguarding the elite varieties and germplasm. DNA fingerprinting have advantages in identifying varieties and are free from environmental impact, compared with customary field inspection (Tommasini et al., 2003). Molecular markers, especially SNPs, are widely used for cultivar identification in many crops (Yoon et al., 2007; Jung et al., 2010; Ganopoulos et al., 2013). In the present study, we have investigated the use of KASP assays for varietal identification of broccoli. Though we have developed 100 KASP assays, to make the fingerprinting cost effective, 28 KASP markers were chosen based on high PIC value and high discriminating power. The fingerprinting result showed that 28 KASP markers are sufficient to distinguish the 372 broccoli accessions, and these markers are considered as core markers for fingerprinting (Supplementary Table 3). The SNP genotype of each broccoli accession was used to generate a corresponding 2D barcode using an online tool (see text footnote 2) and could be used as a reference genetic barcode for each genotyped broccoli accession. This barcode contained fingerprinting information of a large collection (372) of broccoli cultivars in China and can ensure the authenticity of varieties. As SNPs are bi-allelic, allele identification is always comparable among different genotyping platforms. The broccoli SNP barcode database constructed in this study will allow several broccoli breeding groups working in China to use this set of SNPs for varietal identification.

Furthermore, the KASP markers developed in our study can be utilized in genetic purity of the broccoli F_1_ hybrids. For hybrid purity, three to five KASPs will be adequate to ascertain the genetic purity. The markers can be selected from the 100 KASPs according to the parental genotypes of a specific hybrid of broccoli.

## Conclusion

This study provides comprehensive information about the genetic diversity of broccoli accessions in China based on a large population of 372 broccoli accessions. Genome-wide SNPs were discovered by WGS of 23 diverse broccoli genotypes, and the SNPs were converted into GBTS and KASP panels for genotyping of a large set of broccoli accessions. Evaluation of genetic diversity demonstrated that low to moderate genetic diversity prevails in broccoli cultivars widespread in China, and few accessions are in close relationship. This indicated the prevalence of a narrow genetic base among broccoli accessions and the need to broaden the gene pool by adding diverse genotypes into the breeding program. Phylogenetic and PCA analyses based on 1,167 SNPs revealed the presence of two groups but did not show strong groupings. The STRUCTURE results also suggested the presence of two subpopulations with weak genetic structures. The population differentiation was found to be low indicating extensive gene flow. A set of 28 KASP markers was chosen for DNA fingerprints of the broccoli accessions for varietal identification. The SNP genotype of each broccoli accession was used to generate a 2D barcode containing the fingerprinting information of a large collection (372) of broccoli accessions in China. The KASP markers developed in our study could also be used for seed authenticity and purity evaluation of broccoli cultivars. To our knowledge, this is the first study to measure diversity and population structure of a large collection of broccoli in China and also the first application of GBTS and KASP techniques in broccoli for genetic studies. The information generated in the present study will assist in the selection of suitable genotypes for breeding in developing new cultivars as well as physiological and molecular studies in broccoli.

## Data Availability Statement

The datasets presented in this study can be found in online repositories. The names of the repository/repositories and accession number(s) can be found below: https://www.ncbi.nlm.nih.gov/, PRJNA681704.

## Author Contributions

HG designed the experiments, supervised the study, and revised the manuscript. YS and RS analyzed the experimental data and wrote the manuscript. JW and ZZ collected the accessions and selected the core germplasms for re-sequencing. HY, XS, and SL designed the KASP markers and performed the research. All authors discussed the results and contributed to the final manuscript.

## Conflict of Interest

The authors declare that the research was conducted in the absence of any commercial or financial relationships that could be construed as a potential conflict of interest.

## Footnotes

1 www.kbioscience.co.uk

2 www.cli.im

## Supplementary Material

Supplementary Figure 1 Bar plot of the STRUCTURE result.

Supplementary Figure 2 Percentage of molecular variance in the population.

Supplementary Figure 3 Barcode of a representative variety of broccoli.

Supplementary Table 1 Phenotype of the 23 whole-genome sequencing genotypes.

Supplementary Table 2 Information of the GBTS and KASP markers used in this study.

Supplementary Table 3 Genotyping data of 372 broccoli accessions using GBTS and KASP markers.

Supplementary Table 4 The MAF, PIC value, gene diversity, and observed heterozygosity of the markers.

Supplementary Table 5 Genetic distance across the 372 broccoli accessions.

Supplementary Table 6 Mean and standard deviations of the logarithm of the probability of data for different number of sub population (*K*) tested in broccoli.

Supplementary Table 7 AMOVA depicting the genetic variation among and within five subpopulations of broccoli.

Supplementary Table 8 Results of pairwise population Fst analysis when *K* = 5.
