# Supplementary figures and images for: Development of GBTS and KASP Panels for Genetic Diversity, Population Structure, and Fingerprinting of a Large Collection of Broccoli (Brassica oleracea L. var. italica) in China
Source: Front Plant Sci. 2021 Jun 4;12:655254. doi: 10.3389/fpls.2021.655254 (PMC8213352; doi:10.3389/fpls.2021.655254)

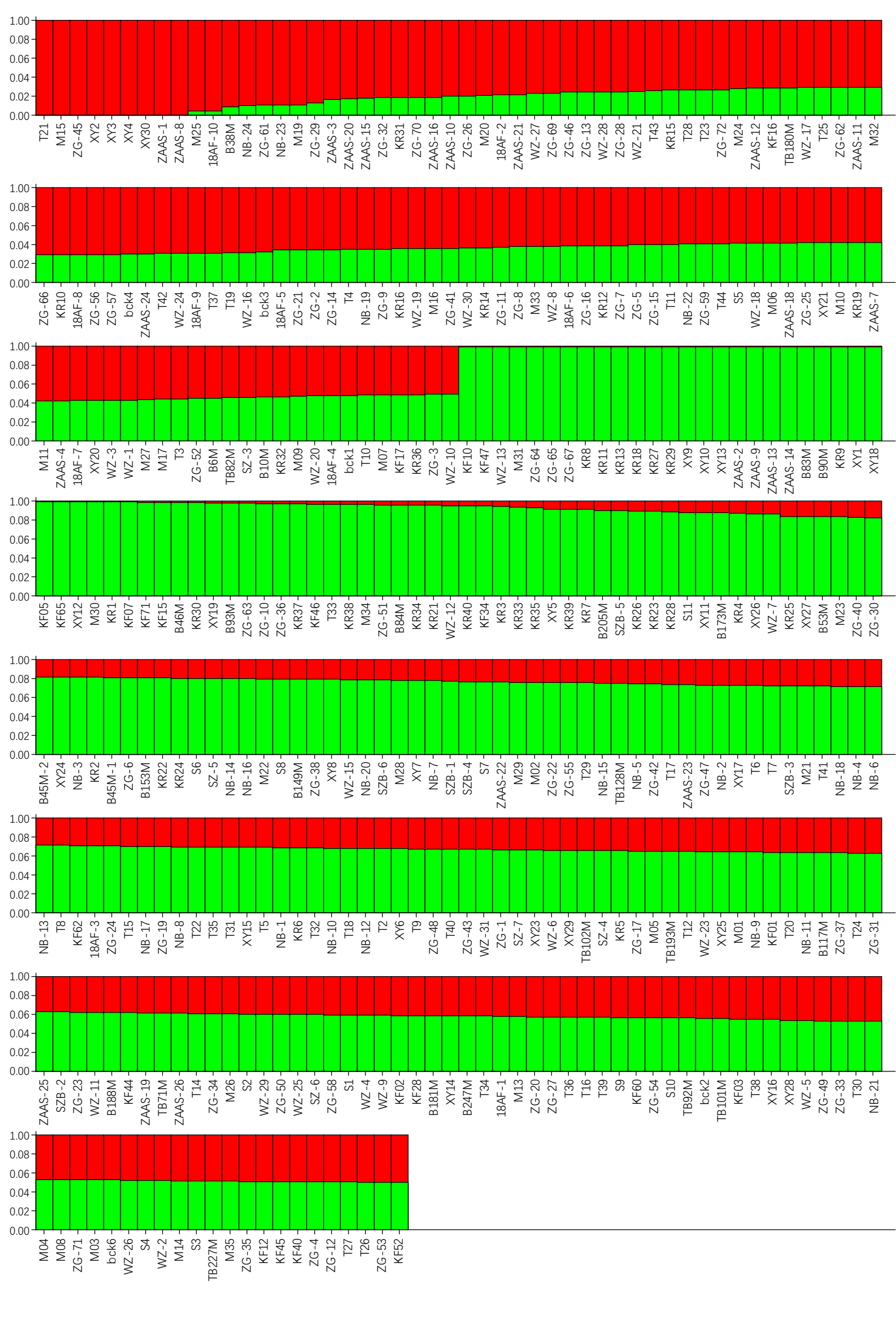

Supplement: Supplementary Figure 1 — Bar plot of the STRUCTURE result. [file Data_Sheet_1.zip › Supplementary Figure 1.PDF]

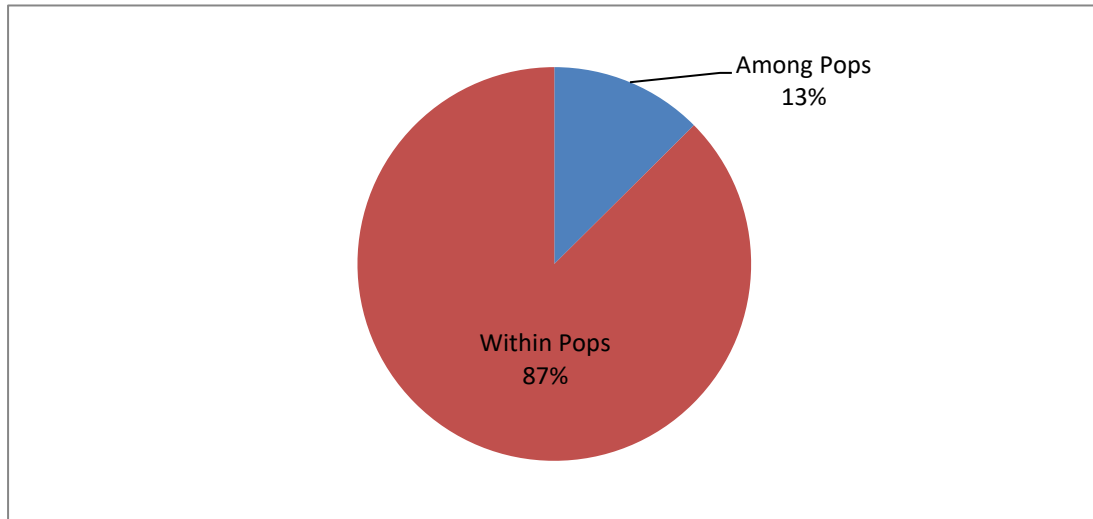

**Supplementary Figure 2.** Percentage of molecular variance in the population

Supplement: Supplementary Figure 1 — Bar plot of the STRUCTURE result. [file Data_Sheet_1.zip › Supplementary Figure 2.PDF]

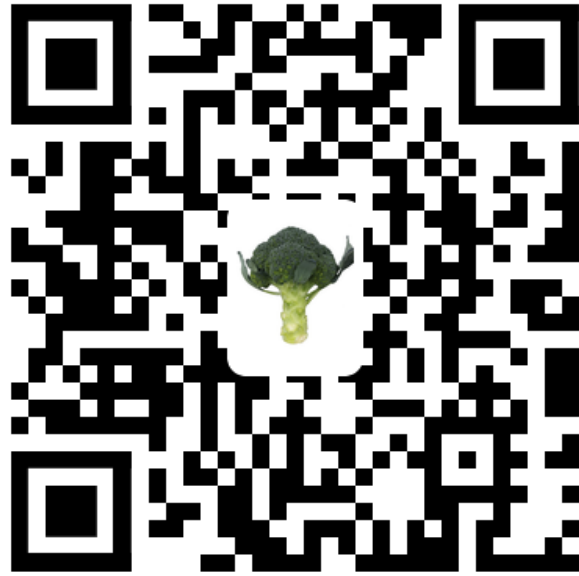

ZAAS-2

**Supplementary Figure 3.** Barcode of a representative variety of broccoli (ZAAS-2).

Supplement: Supplementary Figure 1 — Bar plot of the STRUCTURE result. [file Data_Sheet_1.zip › Supplementary Figure 3.PDF]
